# Loss of Cdx2 Expression in Primary Tumors and Lymph Node Metastases is Specific for Mismatch Repair-Deficiency in Colorectal Cancer

**DOI:** 10.3389/fonc.2013.00265

**Published:** 2013-10-11

**Authors:** Heather Dawson, Viktor H. Koelzer, Anne C. Lukesch, Makhmudbek Mallaev, Daniel Inderbitzin, Alessandro Lugli, Inti Zlobec

**Affiliations:** ^1^Department of Clinical Pathology, Institute of Pathology, University of Bern, Bern, Switzerland; ^2^Translational Research Unit, Institute of Pathology, University of Bern, Bern, Switzerland; ^3^Department of Visceral and Transplantation Surgery, Insel Hospital, Bern University Hospital, Bern, Switzerland; ^4^University Clinic for Visceral Surgery and Medicine, Tiefenau Hospital, Bern, Switzerland

**Keywords:** colorectal cancer, Cdx2, mismatch repair, microsatellite instability

## Abstract

**Background:** Approximately 20% of all colorectal cancers are hypothesized to arise from the “serrated pathway” characterized by mutation in *BRAF*, high-level CpG Island Methylator Phenotype, and microsatellite instability/mismatch repair (MMR)-deficiency. MMR-deficient cancers show frequent losses of Cdx2, a homeodomain transcription factor. Here, we determine the predictive value of Cdx2 expression for MMR-deficiency and investigate changes in expression between primary cancers and matched lymph node metastases.

**Methods:** Immunohistochemistry for Cdx2, Mlh1, Msh2, Msh6, and Pms2 was performed on whole tissue sections from 201 patients with primary colorectal cancer and 59 cases of matched lymph node metastases. Receiver operating characteristic curve analysis and Area under the Curve (AUC) were investigated; association of Cdx2 with clinicopathological features and patient survival was carried out.

**Results:** Loss of Cdx2 expression was associated with higher tumor grade (*p* = 0.0002), advanced pT (*p* = 0.0166), and perineural invasion (*p* = 0.0228). Cdx2 loss was an unfavorable prognostic factor in univariate (*p* = 0.0145) and multivariate [*p* = 0.0427; HR (95% CI): 0.58 (0.34–0.98)] analysis. The accuracy (AUC) for discriminating MMR-proficient and – deficient cancers was 87% [OR (95% CI): 0.96 (0.95–0.98); *p* < 0.0001]. Specificity and negative predictive value for MMR-deficiency was 99.1 and 96.3%. One hundred and seventy-four patients had MMR-proficient cancers, of which 60 (34.5%) showed Cdx2 loss. Cdx2 loss in metastases was related to MMR-deficiency (*p* < 0.0001). There was no difference in expression between primary tumors and matched metastases.

**Conclusion:** Loss of Cdx2 is a sensitive and specific predictor of MMR-deficiency, but is not limited to these tumors, suggesting that events “upstream” of the development of microsatellite instability may impact Cdx2 expression.

## Introduction

Colorectal cancer is a heterogeneous disease at the clinical, histopathological, and molecular level (1). Several molecular classifications of colorectal cancer based on features such as chromosomal instability, point mutations (*APC*, *KRAS*, *BRAF*), microsatellite instability (MSI), and CpG island methylation have been proposed (2–4). It is now generally accepted that approximately 20% of all colorectal cancers arise from serrated adenomas that have undergone a series of genetic changes (5). In the earliest phase of this “serrated pathway” it is hypothesized that mutational activation of *BRAF* leads to an initial burst in proliferation within the normal colonic epithelium followed by p16-induced cell senescence (oncogene-activated senescence) (6, 7). Escape from senescence would be achieved by methylation of p16INK4A, loss of p53 function, or silencing of insulin-like growth factor binding protein 7 (IGFBP7). Responsible for this silencing is the CpG Island Methylator Phenotype (CIMP), a state of aberrant methylation of promoter region CpG islands associated with transcriptional inactivation of tumor suppressor genes (8). These changes lead to the development of sessile serrated adenomas (SSA) that may eventually progress to colorectal cancers (4).

Importantly, among the relevant tumor suppressor genes frequently silenced by CIMP is *MLH1*, a critical gene involved in DNA mismatch repair (9, 10). When hypermethylated, *MLH1* contributes to the development of MSI, a feature observed in 15% of all cases. Defects in the DNA mismatch repair system can be observed by immunohistochemistry for microsatellite instability/mismatch repair (MMR) proteins, such as Mlh1, Msh2, Msh6, and Pms2 (11, 12) with negativity in any one of these proteins a sign of MMR-deficiency.

Interestingly, some studies have observed that MMR-deficient colorectal cancers show a frequent loss of Cdx2, a tumor suppressor gene and homeodomain transcription factor that functions to regulate intestinal epithelial cell differentiation (13–15). Reduced Cdx2 expression has additionally been associated with increased migration and invasion of cancer cells and may play a role in the epithelial mesenchymal transition (EMT) by disrupting WNT pathway signaling (16–21).

The aim of this study is to determine the predictive value of Cdx2 expression for MMR-deficiency, the association with clinicopathological features and patient survival as well as to investigate changes in Cdx2 expression between primary cancers and matched lymph node metastases.

## Patients and Methods

### Patients

The patient cohort consisted of 201 non-consecutive patients treated at the Visceral and Transplantation Surgery department the Insel Hospital in Bern, Switzerland between 2002 and 2011. Gender and age information was available for all patients. Histopathology was systematically re-reviewed. TNM staging was performed in accordance with the seventh edition of the AJCC/UICC staging manual. Clinical metastasis staging (cM) information was available for 190 patients. Lymphatic, venous, and perineural invasion could be reviewed on a majority of cases. Information on adjuvant therapy was available for 197 patients and survival time for 93 patients. No patients received neoadjuvant therapy. Median overall survival time was 54.6 months.

### Specimen characteristics

Formalin fixed (10% neutral buffered formalin) paraffin-embedded tumor blocks were retrieved from the Institute of Pathology, University of Bern, Switzerland. One representative tumor block of primary cancer and lymph node metastases was identified for immunohistochemistry. Ethical consent was obtained from the local ethics commission for both groups.

### Immunohistochemistry

Immunohistochemistry was carried out on whole tissue sections, cut at 4 μm, for all primary colorectal cancers and lymph nodes (Cdx2, Mlh1, Msh2, Msh6, and Pms2). Negative controls were tested with omission of the primary antibodies. An automated Bond III instrument was used along with the following antibodies and protocols: Cdx2, Leica-Novocastra, NCL-Cdx2, 1:200, Tris 95°, 30 min; MLH1, Leica-Novocastra, NCL-MLH1, 1:200, Tris 95°, 30 min; MSH2, Leica-Novocastra, NCL-MSH2, 1:200, Tris 95°, 30 min; MSH6, Leica-Novocastra, MSH6-L-CE, 1:1600, Tris 95°, 30 min; PMS2, Leica-Novocastra NCL-L-PMS2, 1:75, Tris 95°, 30 min. For Cdx2 expression, the percentage of positive tumor cells was estimated. For MMR proteins, any tumor cell expression was defined as positivity for that marker. MMR-deficiency was assigned to cases showing loss of any of the four proteins. Since information on family history was unavailable, no attempt was made to further subdivide patients into Lynch syndrome or sporadic MSI.

### Statistics

The association between Cdx2 expression as continuous variable and MMR status (proficient versus deficient) was investigated using simple logistic regression analysis. Odds ratio (OR) and 95% confidence intervals (CI) were used to determine effect size. The area under the receiver operating characteristic (ROC) curve (AUC) was used to determine the discriminatory ability of Cdx2 expression for MMR-deficiency, with values closer to 1.0 indicating a better discrimination. Cutoffs for Cdx2 focal and diffuse expression were also assessed by ROC curve analysis, by selecting the point on the curve giving the highest sensitivity and specificity for MMR-deficiency. For the association with age, a Wilcoxon’s Test was used and to test the difference in expression between tumor and lymph node, a Wilcoxon’s Signed Rank Test for matched pairs. Univariate survival analysis was performed using the log-rank and Wilcoxon’s tests. Multivariable survival analysis was carried out using Cox regression analysis, with “loss” of Cdx2 used as a baseline. Hazard ratios and 95% CI were used to determine the effect of Cdx2 expression on overall survival. *p*-Values <0.05 were considered statistically significant. All analyses were carried out using SAS V9.2 (The SAS Institute, Cary, NC, USA).

## Results

### Patient characteristics

**Figure 1:**
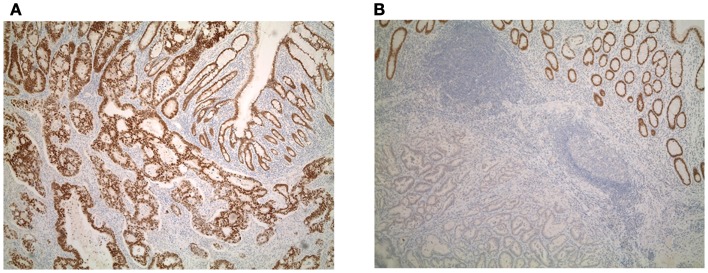
**(A)** Diffuse and **(B)** focal expression of Cdx2 in colorectal cancers.

Patient characteristics are shown in Table 1. Whole tissue sections from 201 patients were evaluated for Cdx2 expression. Of these, 59 patients had available lymph node metastases that underwent Cdx2 staining as well. Representative photomicrographs are shown in Figures 1A,B.

**Table 1:** **Patient characteristics (*n* = 201) and association with Cdx2 expression in tumor**.

Feature		Frequency *N* (%)	Frequency *N* (%)	Frequency *N* (%)	*p*-Value
			
		Total	Focal Cdx2	Diffuse Cdx2	
Gender (*n* = 201)	Male	125 (62.2)	51 (59.3)	74 (64.4)	0.4655
	Female	76 (37.8)	35 (40.7)	41 (35.7)	
Age (years) (*n* = 201)	Median (range)	72.0 (19–91)	70.9 (19–90)	73 (48–91)	0.0825
Tumor location (*n* = 200)	Left	76 (38.0)	28 (32.9)	48 (41.7)	0.3806
	Rectum	29 (14.5)	12 (14.1)	17 (14.8)	
	Right	95 (47.5)	45 (52.9)	50 (43.5)	
Histological subtype (*n* = 200)	Non-mucinous	162 (81.0)	62 (72.1)	100 (87.7)	0.0053
	Mucinous	38 (19.0)	24 (27.9)	14 (12.3)	
Tumor grade (*n* = 199)	G1–2	140 (70.4)	48 (56.5)	92 (80.7)	0.0002
	G3	59 (29.6)	37 (43.5)	22 (19.3)	
pT (*n* = 201)	pT1–2	47 (23.4)	13 (15.1)	34 (29.6)	0.0166
	pT3–4	154 (76.6)	73 (84.9)	81 (70.4)	
pN (*n* = 200)	pN0	92 (46.0)	32 (37.2)	60 (52.6)	0.0303
	pN1–2	108 (54.0)	54 (62.8)	54 (47.4)	
Metastasis (*n* = 190)	cM0	133 (70.0)	50 (63.3)	83 (74.8)	0.0887
	cM1	57 (30.0)	29 (36.7)	28 (25.2)	
Perineural invasion (*n* = 111)	Absence	97 (87.4)	38 (79.2)	59 (93.7)	0.0228
	Presence	14 (12.6)	10 (20.8)	4 (6.4)	
Venous invasion (*n* = 132)	Absence	59 (44.7)	21 (37.5)	38 (50.0)	0.1534
	Presence	73 (55.3)	35 (62.5)	38 (50.0)	
Lymphatic invasion (*n* = 128)	Absence	32 (25.0)	10 (17.5)	22 (31.0)	0.0809
	Presence	96 (75.0)	47 (82.5)	49 (69.0)	
Adjuvant therapy (*n* = 197)	None	135 (68.5)	48 (57.8)	87 (76.3)	0.0058
	Treated	62 (31.5)	35 (42.2)	27 (23.7)	
Mismatch repair status (*n* = 201)	Proficient	174 (86.6)	60 (69.8)	114 (99.1)	<0.0001
	Deficient	27 (13.4)	26 (30.2)	1 (0.9)	
Overall survival (*n* = 93)	Median (95%CI)	54.6 (28–72)	26.4 (10–61)	68.7 (44–101)	0.0145

### Association of Cdx2 in tumor and lymph nodes with clinicopathological features

**Figure 2:**
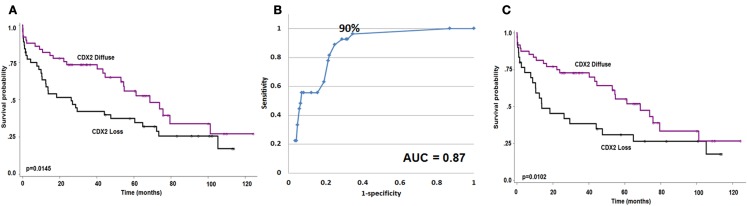
**(A)** Kaplan–Meier survival curve showing effect of Cdx2 expression on overall survival (*n* = 93). **(B)** ROC curve highlighting the strong predictive effect and specificity of loss of Cdx2 expression for MMR-deficiency. **(C)** Kaplan–Meier survival curve showing effect of Cdx2 expression on overall survival in MMR-proficient colorectal cancer patients only (*n* = 78). Wilcoxon’s test.

Focal Cdx2 expression was significantly more frequent in colorectal cancers with mucinous histology (*p* = 0.0053), higher tumor grade (*p* = 0.0002), more advanced pT stage (*p* = 0.0166), with perineural invasion (*p* = 0.0228), and in those receiving adjuvant therapy (*p* = 0.0058). In addition, there was a significant and adverse effect of Cdx2 loss on patient survival (*p* = 0.0145; Figure 2A). This result was maintained in multivariable analysis with pT and pN classifications [*p* = 0.0427; HR (95% CI): 0.58 (0.34–0.98)] but not when clinical metastasis staging was included in the model. Although not statistically significant, possibly due to a smaller number of patients, loss of Cdx2 seemed to occur more frequently in tumors with lymphatic invasion (*p* = 0.0809), and in patients with metastasis (*p* = 0.0887).

Table 2 shows the associations between lymph node expression of Cdx2 and clinicopathological features of the primary cancers. Indeed, only tumor location was linked to loss of Cdx2 expression, which occurs more frequently in the right-side of the colon (*p* = 0.0088). Of the 59 patients with evaluable lymph nodes, information on survival was only available in 26. Loss of Cdx2 in lymph node metastasis was marginally associated with overall survival (*p* = 0.0512).

**Table 2:** **Association of Cdx2 loss in tumor and lymph nodes with clinicopathological features (*n* = 59)**.

Feature		Lymph node metastases (*n* = 59)		
		Cdx2 focal	Diffuse	*p*-Value
Gender (*n* = 59)	Male	9 (64.3)	29 (64.4)	1.0
	Female	5 (35.7)	16 (35.6)	
Age (years) (*n* = 59)	Median (range)	71 (19–87)	74 (30–91)	0.1169
Tumor location (*n* = 58)	Left	1 (7.1)	20 (45.5)	0.0088
	Right	5 (35.7)	4 (9.1)	
	Rectum	8 (57.1)	20 (45.5)	
Histological subtype (*n* = 59)	Non-mucinous	10 (71.4)	34 (75.6)	0.7376
	Mucinous	4 (28.6)	11 (24.4)	
Tumor grade (*n* = 59)	G1–2	7 (50.0)	32 (71.1)	0.3156
	G3	7 (50.0)	13 (28.9)	
pT (*n* = 59)	pT1–2	2 (14.3)	4 (8.9)	0.6204
	pT3–4	12 (85.7)	41 (91.1)	
Metastasis (*n* = 56)	cM0	9 (75.0)	23 (52.3)	0.1585
	cM1	3 (25.0)	21 (47.7)	
Perineural invasion (*n* = 33)	Absence	6 (75.0)	20 (80.0)	1.0
	Presence	2 (25.0)	5 (20.0)	
Venous invasion (*n* = 41)	Absence	2 (18.2)	8 (26.7)	0.7004
	Presence	9 (81.8)	22 (73.3)	
Lymphatic invasion (*n* = 44)	Absence	1 (8.3)	1 (3.1)	0.4757
	Presence	11 (91.7)	31 (96.9)	
Adjuvant therapy (*n* = 59)	None	7 (50.0)	23 (51.1)	0.9421
	Treated	7 (50.0)	22 (48.9)	
Mismatch repair status (*n* = 59)	MMR-proficient	7 (50.0)	43 (95.6)	<0.0001
	MMR-deficient	7 (50.0)	2 (4.4)	

Evaluating the matched lymph nodes and primary colorectal cancers, average expression was 66.7% in lymph nodes and 71.0% in primary tumors. Using a matched pairs analysis, this difference was not significant (*p* = 0.5801).

### Cdx2 expression and mismatch repair status

There was a major significant association between reduced Cdx2 expression and MMR-deficiency. The AUC value for Cdx2 expression in tumor was 0.87 indicating 87% accuracy for discriminating MMR-proficient and – deficient cancers (Figure 2B). The OR (95% CI) was 0.96 (0.95–0.98); *p* < 0.0001. The ROC curve was used as a basis for the identification of an optimal threshold value for considering tumors with “focal” and “diffuse” expression and determined to be 90%. Of the 115 patients with diffuse expression of Cdx2, 114 were MMR-proficient (99.1% specificity) and of the 27 MMR-deficient patients 26 had only focal expression (96.3% negative predictive value). There were 174 patients with MMR-proficient cancers, of which 60 (34.5%) indeed showed loss of Cdx2. Cdx2 loss among patients with MMR-proficient cancers was significantly and unfavorably related to survival (*p* = 0.0102; Figure 2C). Again, in multivariable analysis, Cdx2 loss was associated with worse outcome after adjusting for pT and pN [*p* = 0.0414; HR (95% CI): 0.54 (0.3–0.98)], but not when clinical metastasis stage was added.

The AUC for Cdx2 expression in lymph nodes and MMR status was 0.943 indicating 94% discriminatory ability of the protein. The OR (95% CI) was 0.93 (0.87–0.99); *p* = 0.037. Using the ROC curve for the selection of a threshold value, tumors with <30% staining were considered “focal” and >30% considered “diffuse” for Cdx2 expression. Of the 45 cases with diffusely expressing Cdx2, 43 were MMR-proficient (95.6% specificity), whereas 7/9 MMR-deficient cancers showed focal expression of Cdx2 (77.8% NPV).

## Discussion

The findings of this study suggest that reduced expression of Cdx2 in primary tumors and lymph node metastases is an accurate predictor of MMR-deficiency in colorectal cancer. Moreover, loss of Cdx2 is a poor prognostic factor, even among patients with MMR-proficient cancers.

In a first step, we examined the specificity of Cdx2 for MMR status. The ROC curve for this analysis underlines the major discriminatory power of reduced Cdx2 expression for MMR-deficiency in both colorectal cancers and lymph nodes. Previous reports by our group and others have highlighted similar findings. Using a tissue microarray containing more than 600 patient tissues, Baba and colleagues showed a high specificity of reduced Cdx2 expression for MSI-high colorectal cancers (22). The protein expression of Cdx2 in MMR-proficient versus deficient cancers has been reported at 84 versus 61% on average, again using tissue microarrays (14). Our study goes one step further and uses whole tissue sections for the establishment of both MMR status and Cdx2 expression. Indeed, all MMR-deficient cancers with the exception of one case showed only focal positivity for Cdx2 expression.

**Figure 3:**
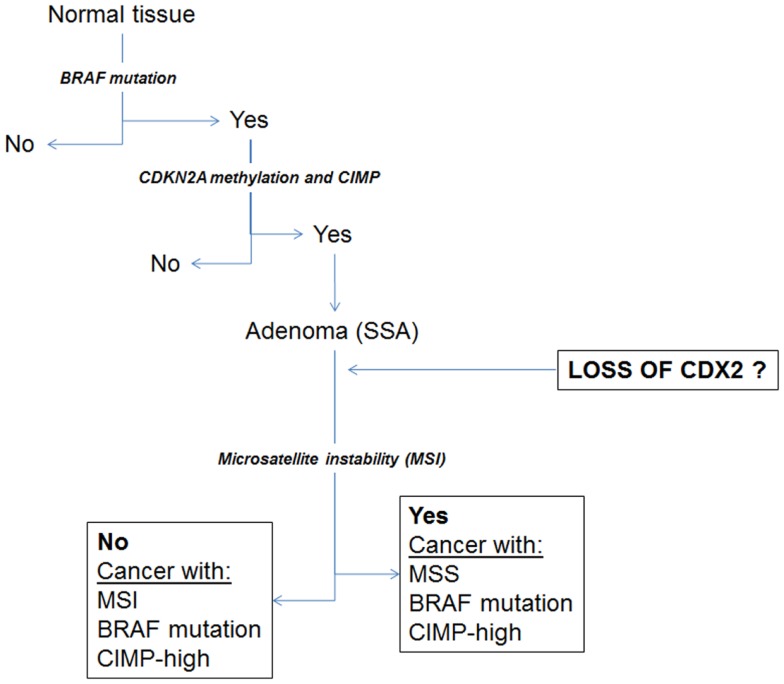
**Schematic diagram illustrating a proposal for the involvement of Cdx2 during progression of cancers through the serrated pathway**.

Despite this observation, a subgroup of MMR-proficient cancers also shows focal positivity for Cdx2. Our hypothesis is that Cdx2 loss may be an important marker of other molecular changes associated with the serrated pathway to colorectal cancer, including *BRAF* mutation and high-level CIMP. Indeed, we could previously show using a cohort of more than 300 patients, that loss of Cdx2 was nearly 100% specific for *BRAF* mutation, and found in 23/24 mutated cases (23). Baba and colleagues as well as Walsh et al. found loss of Cdx2 in *BRAF* mutated tumors and a significantly more frequent number of cases in tumors with CIMP-H (22, 24). Loss of Cdx2 has also been found to be an independent predictor of the CIMP-H phenotype (25). Figure 3 illustrates some of the changes hypothesized to occur during the serrated pathway. We believe that loss of Cdx2 expression occurs prior to the establishment of MSI and only after the development of both *BRAF* mutation and CIMP. Although the evidence fits well for an involvement of Cdx2 in the serrated pathway, whether this molecule is actually functionally involved as a cause rather than a consequence of progression of tumors within this pathway has not yet been established.

Next, we evaluated the association between focal expression of Cdx2 and clinicopathological features. Our results point toward an association of Cdx2 with an array of important and adverse prognostic features including unfavorable overall survival. Our findings are in line with previous work from our group using a single punch tissue microarray showing strong correlations between Cdx2 loss and pT, pN, tumor grade, and vascular invasion on more than 1000 tumors. Baba and colleagues showed similar results of Cdx2 loss with more advanced TNM stage, higher tumor grade, mucinous, or signet ring cell histology (22). These results are in agreement with Choi et al. who show loss of Cdx2 expression associated with advanced Dukes’ stage and more poorly differentiated cancers (26). Unfavorable survival times are reported by several groups upon reduced Cdx2 expression (22, 27). In addition, the predilection for female gender and more right-sided tumor location has also been observed in other studies (22, 28). We also show that the unfavorable impact of Cdx2 is maintained in patients with MMR-deficient cancers.

Thirdly, we evaluated for the first time Cdx2 expression in matched lymph node metastases. We found no differences in expression between lymph nodes and primary colorectal cancers. These results appear to indicate that a further “evolution” leading to loss of Cdx2 after lymph node spread is unlikely.

To conclude, Cdx2 is significantly reduced in patients with MMR-deficient colorectal cancers, but is not limited to these tumors. It is an unfavorable prognostic factor, even among patients with MMR-proficient cancers. Taken together with previous reports on *BRAF* and CIMP, we hypothesize that Cdx2 loss may play an early role in the progression of cancers arising through the serrated pathway.

## Conflict of Interest Statement

The authors declare that the research was conducted in the absence of any commercial or financial relationships that could be construed as a potential conflict of interest.
